# Reactive stepping with functional neuromuscular stimulation in response to forward-directed perturbations

**DOI:** 10.1186/s12984-017-0266-6

**Published:** 2017-06-10

**Authors:** Alexander J. Hunt, Brooke M. Odle, Lisa M. Lombardo, Musa L. Audu, Ronald J. Triolo

**Affiliations:** 1 Department of Biomedical Engineering, Case Western Reserve University, Cleveland, OH 44106 USA; 2 Department of Mechanical and Materials Engineering, Portland State University, 1930 SW 4th Ave, Portland, OR 97201 USA; 3 Department of Veterans Affairs, Advanced Platform Technology Center, Louis Stokes Cleveland VA Medical Center, Cleveland, OH 44106 USA; 4 Department of Orthopedics, Case Western Reserve University, Cleveland, OH 44106 USA

**Keywords:** Spinal cord injury, Functional neuromuscular stimulation, Neuroprostheses, Standing balance, Reactive stepping

## Abstract

**Background:**

Implanted motor system neuroprostheses can be effective at increasing personal mobility of persons paralyzed by spinal cord injuries. However, currently available neural stimulation systems for standing employ patterns of constant activation and are unreactive to changing postural demands.

**Methods:**

In this work, we developed a closed-loop controller for detecting forward-directed body disturbances and initiating a stabilizing step in a person with spinal cord injury. Forward-directed pulls at the waist were detected with three body-mounted triaxial accelerometers. A finite state machine was designed and tested to trigger a postural response and apply stimulation to appropriate muscles so as to produce a protective step when the simplified jerk signal exceeded predetermined thresholds.

**Results:**

The controller effectively initiated steps for all perturbations with magnitude between 10 and 17.5 s body weight, and initiated a postural response with occasional steps at 5% body weight. For perturbations at 15 and 17.5% body weight, the dynamic responses of the subject exhibited very similar component time periods when compared with able-bodied subjects undergoing similar postural perturbations. Additionally, the reactive step occurred faster for stronger perturbations than for weaker ones (*p* < .005, unequal varience *t*-test.)

**Conclusions:**

This research marks progress towards a controller which can improve the safety and independence of persons with spinal cord injury using implanted neuroprostheses for standing.

## Background

Neuroprostheses employing functional neuromuscular stimulation (FNS) are effective tools for increasing the personal mobility of persons with a spinal cord injury (SCI) [1, 2]. These systems apply small electrical currents to the motor nerves, which cause the otherwise paralyzed muscles to contract and apply forces to actuate the limbs of the body. Electrically activating the paralyzed lower extremity and trunk muscles of persons with thoracic level SCI has enabled system recipients to regain the ability to stand, perform transfers, and walk short distances [3]. Neuroprostheses that restore or enhance standing function after paralysis are not yet self-stabilizing and require users to exert forces on an external device with their upper extremities (UE) to maintain standing balance. To improve functionality of these prostheses, increase safety, and reduce reliance on the UE, neuroprostheses must be able to react to environmental perturbations and modulate stimulation to maintain an upright posture.

Stabilizing the body involves a complex interaction between mechanical constraints, movement and sensory strategies, orientation in space, control of dynamics, and cognitive processing [4]. Developing balance controllers for a neuroprosthesis is exceptionally difficult because all of these aspects are significantly altered by SCI and the limitations inherent with electrical stimulation. There are additional constraints on the mechanical system such that only a small subset of muscles in the extremities can be activated by existing neuroprostheses. Additionally, the muscles activated by neural stimulation are recruited differently from normal processes in able-bodied individuals, with larger and more fatigable motor units generally first, which results in significant challenges to attaining stable erect postures for prolonged periods of time. Furthermore, SCI can interrupt the flow of information to the brain, effectively disconnecting cognitive processing from the sensory system and the direct perception of the actions of the contracting muscles.

In spite of these challenges, progress is being made toward improving postural control of standing with neuroprostheses. Stabilizing posture without use of the hands or arms in response to internal and external disturbances often occurs from increased joint torques at the hips and ankles [5]. Recent studies have successfully developed and deployed advanced control systems which modulate stimulation to muscles of the lower extremities and trunk in order to effect erect posture recovery without repositioning the feet in response to perturbations on the order of 5–10% body weight (BW) [6–8]. However, this strategy is ineffective for larger perturbations.

When the perturbation is large enough to indicate the potential for the center of mass (COM) to exceed the limits of the base of support (BOS) defined by the area enclosed by the borders of the feet, the intact central nervous system initiates a compensatory step to alter foot placement so as to maintain balance. A number of complex processes are involved in a reactive compensatory step. Depending on the magnitude of the perturbation, this step involves an anticipatory postural adjustment (APA) in which the body’s COM is shifted in the medial-lateral direction between the two legs to near the position of the stance foot. Thereafter the swing leg lifts off the ground and is repositioned ahead of the stance foot [9, 10]. When the magnitude of postural perturbations is increased or is unexpected, the time period for the APA is shortened or even eliminated [11]. For perturbations of more moderate magnitude, an APA response can also be evoked without a step occurring [12]. This suggests that different mechanisms or movement thresholds are in effect during the preparation to step, as opposed to the taking of the actual step.

The current report describes the design and testing of a controller that detected forward-directed disturbances larger than 10% BW applied to a subject with SCI and initiated a reactive step via FNS to lengthen the base of support by repositioning the feet into a tandem stance, thus potentially increasing standing stability. The controller utilizes a signal derived from three accelerometers mounted on the subject’s torso and upper leg to detect and characterize the applied perturbations. The processed signal reliably differentiated perturbation size and triggered steps more quickly for larger perturbations by utilizing a biologically inspired multiple threshold technique similar to able-bodied reactions. The completed step has similar latencies to an able-bodied person’s reactive step when comparing reaction time, posture shifting (APA), leg lift, and step completion times.

## Methods

### Subject and FNS control system hardware

The participant was a 59 year-old, male with T4 level sensory incomplete/motor complete (AIS B) paraplegia. He was approximately 175 cm tall and weighed 804 N. The subject signed informed consent forms approved by the Institutional Review Board of the Louis Stokes Cleveland Department of Veterans Affairs Medical Center. He was an active user of an implanted FNS system for the restoration of basic standing function at the time of all experimental data collection. The neuroprosthesis included a surgically implanted 16-channel stimulator-telemeter device (IST) [13] and an implanted 8-channel receiver-stimulator device (IRS) [1]. The implanted pulse generators delivered current-controlled, asymmetrical, charge-balanced stimulating waveforms to the targeted motor nerves via intramuscular [13] or spiral nerve cuff [14] electrodes. Pulse amplitudes (1.4, 2.0, 2.1, 8, 14 or 20 mA) were set on a channel-by-channel basis while pulse duration (0–250 μsec) and frequency (0–30 Hz) were modulated independently on a pulse-by-pulse basis on each channel to achieve the desired motion. A wearable external control unit (ECU) [15] delivered power and command signals to the implanted pulse generators via close-coupled inductive links maintained by wire coils taped to the skin over the stimulators. The ECU has a user interface consisting of command buttons and an LCD screen on the enclosure and a re-chargeable lithium ion battery. It coordinated the delivery of temporal patterns of stimulation through all 24 channels simultaneously.

Stimulation was applied via intramuscular electrodes implanted near innervation points of the following muscle groups bilaterally: semimembranosus/hamstrings (HS, hip extension), posterior portion of adductor magnus (PA, hip adduction), gluteus maximus (GM, hip extension), erector spinae (ES, trunk extension), quadratus lumborum (QL, trunk lateral bending) iliacus/psoas major (IL, hip flexion) and right leg muscle groups: sartorius (ST, hip and knee flexion), tensor fasciae latae (TF, hip flexion and internal rotation), and gluteus medius (ME, hip abduction). To maximize recruitment and stimulated stance limb hip extension torque production, additional intramuscular electrodes and stimulus channels were dedicated to the left HS and left GM. Spiral nerve cuff electrodes were surgically installed around the femoral nerves in the proximal thighs to selectively activate the three uniarticular vasti muscles of the quadriceps (VS, knee extension) while avoiding recruitment of the biarticulate rectus femoris, which induces hip flexion that compromises erect neutral standing. Spiral nerve cuffs were also deployed below the knee on the branches of the fibular nerve innervating the tibialis anterior (TA, ankle dorsiflexion), and tibial nerve innervating the gastrocnemius/soleus (GS, ankle plantarflexion).

Real-time control of the 24 channel implanted neural stimulation system was implemented with custom software developed to run in MATLAB®/SIMULINK® and the xPC Target™ toolbox (Mathworks, Inc., Natick, MA). A Windows® (Microsoft, Inc., Redmond, WA) host computer was utilized to build customized applications, while a dedicated target computer with a Pentium Dual-Core 3 GHz microprocessor (Intel, Inc., Santa Clara, CA) with 2 GB of RAM was responsible for running the applications in real-time. The host and target communicated via TCP/IP protocol. Data were acquired using an NI PCI-6071E board (National Instruments, Inc., Austin, TX), and all real-time controller parameter and stimulation updates were fixed at 40 Hz. Stimulus values for upright standing were determined by clinical observation whereby the subject exhibited ample knee and trunk extension to achieve an erect posture without discomfort. Standing stimulation values are listed in Appendix. The inter-pulse interval during standing stimulation was fixed at 50 ms (20 Hz) for all experiments.

To ensure the subject’s ability to comfortably and safely maintain near erect bipedal standing, nonzero minimum levels of stimulation pulse width were applied continuously based on clinical observation for the channels activating the following core muscle groups for knee and hip extension bilaterally: VI, HS, and GM. Activation of these muscles remained constant during the entire experimental procedure except to complete swing on the non-stance limb during reactive stepping.

### Determination of stepping control parameters

During the subject’s rehabilitation with the implanted neuroprosthesis, a clinical stepping pattern was developed for home use with a walker. This pattern was developed by trial and error of stimulation magnitudes and timing to produce forward steps. Initial parameters were informed by experimentally mapping activation magnitudes of the neuroprosthesis to joint torques and a qualitative understanding of the kinematics of able-bodied adult stepping. Timing and magnitudes were then adjusted to ensure proper ground clearance with a comfortable stepping behavior. For example, to flex the hip for swing the GM, HS, and PA were turned off and IL, ST, and TFL muscles were turned on. Stimulus parameter values ramped up or down over short time frames rather than abruptly changing from one value to another. Clinical stepping had an approximate step time of 2 s from initiation to a return to double leg support and resumption of static standing with stimulation as described above. Based on preliminary simulations with a subject-specific musculoskeletal model and able-bodied human data, the total time required for effective reactive stepping must be less than 1 s [16]. To develop an effective way to reduce the stepping time, a swing stimulus pattern consisting of 2 phases was developed: one pattern for leg flexion and a different one for leg extension. Stimulation pulse widths in each phase, and the length of each phase were manually adjusted to achieve a step with the right leg while the subject remained standing in place. Additionally, the inter-pulse interval was reduced to 33.3 ms (30 Hz) during step stimulation to reduce the time to muscle contraction after stimulation onset. The chosen pattern was one that produced a fast step with sufficient ground clearance. The final pattern, shown in Table 1, was saved as a pre-programmed pattern for use in the reactive stepping experiments. Stimulation time for the leg flexion phase was set at 500 ms and stimulation for the leg extension phase was set at 400 ms for a total step stimulation pattern of 900 ms.

**Table 1:** Stepping parameters for the four states of the reactive stepping controller

Muscle	Standing Activation (R/L %)	Posture Shift Activation (R/L %)	Flexion Activation (R/L %)	Extension Activation (R/L %)
Vasti (VS)	100/100	100/100	0/100	100/100
Semimembranosus/hamstrings (HS)	100/100	100/100	0/100	0/100
Gluteus maximus (GM)	100/100	100/100	0/100	0/100
Posterior adductor magnus (PA)	0/100	0/100	0/100	0/100
Iliacus/psoas major (IP)	0/0	0/0	100/0	75/0
Gastrocnemius/soleus (GS)	0/0	100/100	0/0	0/0
Tibialis anterior (TA)	0/0	0/0	100/50	0/50
Quadratus lumborum (QL)	0/0	0/0	0/0	0/0
Erector spinae (ES)	0/0	0/0	0/0	0/0
Gluteus medius (ME)	0/−	100/−	100/−	100/−
Sartorius (SR)	0/−	0/−	100/−	0/−
Tensor fasciae latae (TF)	0/−	0/−	100/−	0/−

Activation is represented in terms of percent of saturation stimulation

### Laboratory perturbation testing

**Fig. 1:**
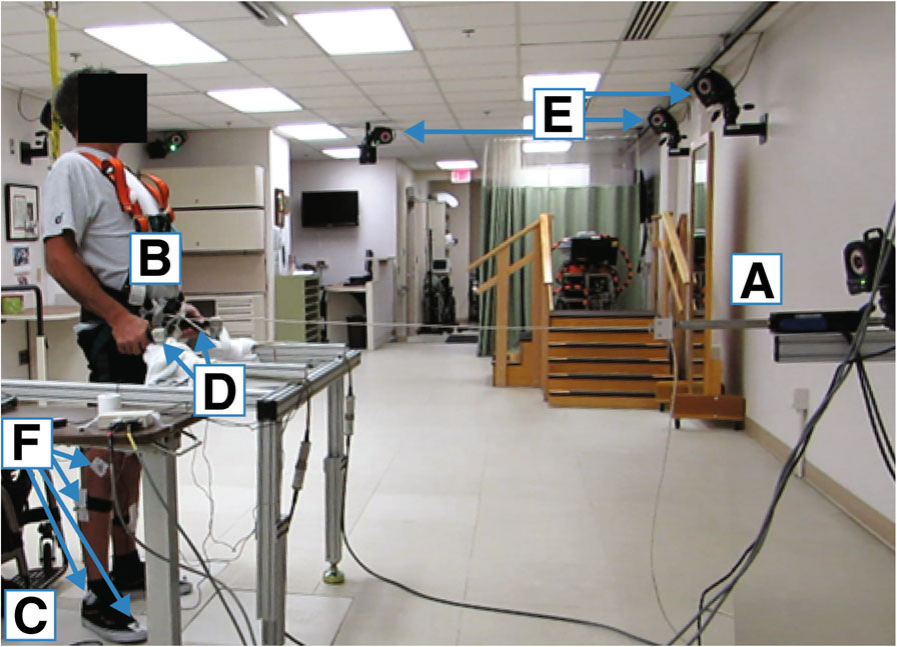
Experimental setup. Linear actuator (**a**) pulls on cable attached to a weight lifter’s belt at the waist (**b**). The subject stands on force plates (**c**) and mantains balance by holding onto the instrumented handles attached to the fixed support structure (**d**). Vicon cameras (**e**) capture motion through markers attached to the legs and upper body (**f**). Accelerometer data processing and control are performed on a realtime target computer (*off image*)

Experiments were conducted with the subject in a neutral, bipedal stance (Fig. 1), with each foot on a force platform (OR6–6-1000, Advanced Mechanical Technology, Inc., Watertown, MA). The subject balanced himself against postural disturbances by applying corrective loads with his upper extremities (UE) on instrumented walker handles with six-axis load cells (MCW-500, Advanced Mechanical Technology, Inc., Watertown, MA) attached to a customized support device composed of aluminum framing (80/20®, Inc., Columbia City, IN). Analog data from the force plates and load cells were sampled at 400 Hz. The 3D positions of body segments were measured with respect to a globally fixed reference frame approximately aligned to anatomical anterior-posterior and medial-lateral planes of the subject using a VICON® MX digital motion capture system (Vicon Motion Systems and Peak Performance, Inc., Oxford, UK). Reflective markers were placed on anatomical landmarks on the legs and trunk according to a subset of the *PlugInGait* marker set (C7 vertebra, clavicle, sacrum and bilateral shoulder, anterior superior iliac spine, posterior superior iliac spine, thigh, knee, tibia, ankle, heel, and toe). Motion capture data were sampled at 100 Hz. For safety, the subject wore a harness (McMaster-Carr, Inc., Elmhurst, IL) connected to a structurally reinforced overhead hook via a lanyard (Guardian Fall Protection, Inc., Kent, WA).

External perturbations were systematically applied as anteriorly-directed force-pulses by an electromagnetic linear actuator (STA2506, Copley Controls, Inc., Canton, MA). The actuator was programmed to pull the subject forward via a nylon cable attached to a weight lifting belt placed just above the participant’s waist. The actuator was mounted on a custom aluminum support structure (80/20®, Inc., Columbia City, IN) rigidly fixed to the wall directly in front of the subject.

Postural perturbations were applied to the subject while standing with his clinical standing stimulation pattern. Distinct disturbance magnitudes were applied at 2, 5, 10, 15, and 17.5% BW. All disturbances were 300 msec in duration. The maximum magnitude of 17.5% BW was determined as the upper limit at which the subject felt comfortable resisting using UE effort alone. Perturbations were applied at random intervals ranging from 2 to 5 s after a ready assent was given by the subject. There were approximately 20 s between pulls in a set, allowing the subject to return the stepping foot to the force plate and resume an erect bipedal stance in preparation for the next perturbation. Experiments were performed over two sessions separated by 4 months. Each session consisted of several rounds of standing for 5–10 min after which the subject rested for 10–20 min before commencing the next round. In the first session, the first 3 rounds consisted of determining the stepping parameters while the final 2 rounds consisted of 12 pulls; 2–3 pulls were performed at each of the 5 distinct disturbance magnitudes and observations were used to make further refinements to the controller parameters. The second session consisted of 1 round of 17 pulls, and a second round of 14 pulls. The first round was performed at 15% BW and the second was at 17.5% BW. Data for step timing and step magnitudes were collected in each trial.

**Fig. 2:**
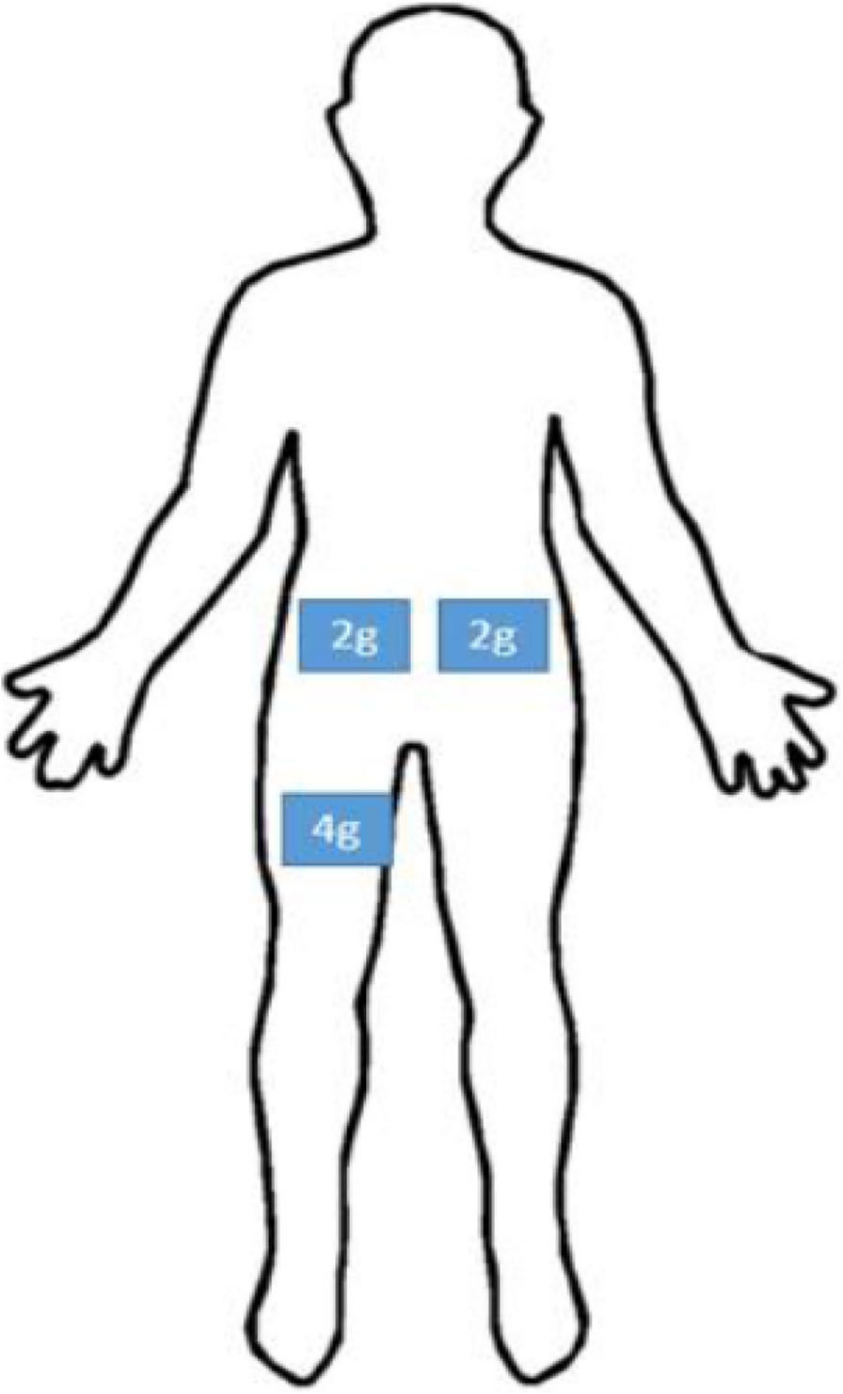
Locations of body-mounted accelerometers onthe subject

For detecting body responses to the perturbations, three triaxial accelerometers (Crossbow Technology, Milipitas, CA) were taped on the subject. Two were placed on either side of the umbilicus (max 2 g reading), and one placed on the right thigh (max 4 g reading), as depicted in Fig. 2. Sensor positions were chosen to coincide with estimated location of the whole body center of mass. Analog signals from the accelerometers were collected and processed by the real-time controller at 40 Hz.

### Reactive stepping controller design

**Fig. 3:**
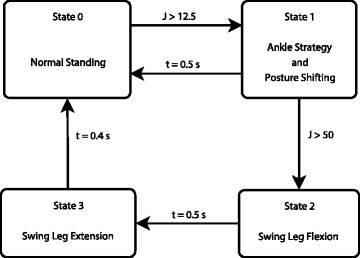
Diagram of the finite state reactive stepping controller. The controller switches between clinical baseline standing stimulation and the stepping pattern when the processed signal crosses distinct thresholds or a timeout occurs

The reactive stepping controller consisted of a finite state machine in which switching was performed either by a processed accelerometer signal or time outs. The overall control scheme is depicted in Fig. 3. Throughout the experiment, the right leg was the swing leg while the left was the support leg.

The present study and controller assumed a forward-directed perturbation; accelerometer orientation was eliminated from the control by calculating the magnitude of the jerk for each axis on each accelerometer and summing them together. Noise was reduced by passing the result through a 2nd order lowpass Butterworth filter with a cuttoff frequency of 0.2. The processed jerk signal was calculated as:

1 $$ {J}_t= butter\left(\sum_{i=1}^3\sum_{j=1}^3\frac{\left|{a}_{i, j, t}-{a}_{i, j, t-1}\right|}{\Delta t}\right) $$

where *a*_*i* , *j* , *t*_ is the acceleration of the *i*th axis of the *j*th accelerometer at time *t* and ∆*t* = 0.025 *s*.

**Fig. 4:**
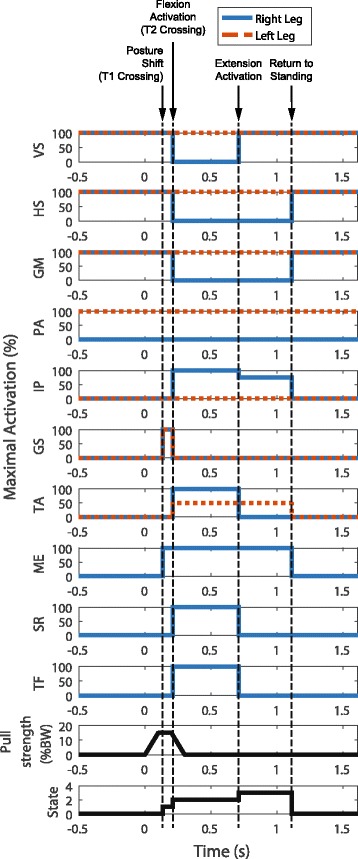
Stimulation patterns, perturbation and controller states before and after application of anterior force pulses were applied (*t* = 0). Muscle abbreviations are: Vasti (VS), Semimembranosus/hamstrings (HS), Gluteus maximus (GM), Posterior adductor magnus (PA), Iliacus/psoas major (IP), Gastrocnemius/soleus (GS), Tibialis anterior (TA), Gluteus medius (ME), Sartorius (SR), Tensor fasciae latae (TF)

The four states of the reactive stepping controller were (0) clinical baseline standing, (1) ankle perturbation rejection and pre-step posture shifting, (2) swing leg flexion, and (3) swing leg extension. An overview of the state machine is depicted in Fig. 3. The activations for the muscles in each of these states are listed in Table 1. During the clinical baseline standing state, the bilateral vasti group (VS), semimembranosus/hamstrings (HS), gluteus maximus (GM), and the left posterior adductor magnus (PA) were activated at maximal values as shown in column 1 of Table 1. Clinical baseline standing was active at all times except during perturbation rejection control. The time course of the stimulation pattern before and after the onset of a pull, along with associated changes in controller state, can be seen in Fig. 4. Maximal stimulation values were chosen empirically for each muscle ahead of time and were the lowest of three options: maximum stimulation the subject finds comfortable, stimulation at which no more strength is observed under higher pulse widths, or the maximum stimulation the controller unit can provide. These maximum stimulation values are listed in Appendix.

**Fig. 5:**
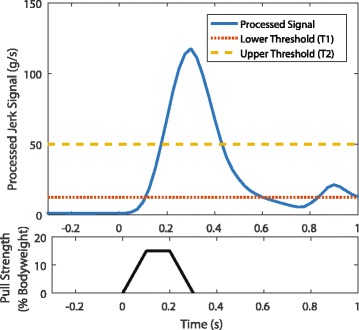
Combined jerk signal derived from the three body mounted accelerometers (Eq. 1) at 15% BW strength pull. Average time to cross the thresholds at 15% BW was 133 ms for the lower threshold (T1) and 210 ms for the higher threshold (T2)

During the ankle perturbation rejection and pre-step posture shifting state (State 1), the stimulus levels for clinical baseline standing remained active with the addition of both left and right gastrocnemius/soleus (GS) to resist the perturbation while the right gluteus medius (ME) was activated to shift weight off of the right foot. This state was activated when the combined jerk derived from the processed accelerometer data (Eq. 1) crossed a low threshold (T1) and ended when either swing leg flexion commenced with the crossing of a higher threshold (T2) or if the higher threshold was not achieved after a pre-set time interval (500 ms). A typical signal created at a 15% BW pull and the threshold crossings are displayed in Fig. 5.

Thresholds T1 and T2 were empirically determined through extensive tuning with the subject in the loop. T1 was set above clinical baseline standing and voluntary movements and T2 was set between the signal values for body perturbations above 5% and below 10% BW. This ensured that smaller perturbations caused an ankle perturbation rejection response but do not cause a stepping response. The values for T1 and T2 were 12.5 g/s and 50 g/s respectively, where g is acceleration due to gravity.

During the swing leg flexion state (State 2), stimulation for the left leg muscles remained at clinical baseline standing values with the addition of half-strength left tibialis anterior (TA) activation to produce ankle dorsiflexion that pulled the body forward. Activation of the right leg QU, HS, and GM was removed while activation of the right ME was maintained. Simultaneously, the right hip flexors iliacus/psoas major (IP), sartorius (SR), and tensor fasciae latae (TF), and the ankle dorsiflexor (TA) are also activated at full strength to flex the leg as quickly as possible. State 2 was activated by a higher acceleration threshold (T2) and was active for 500 ms.

During the swing leg extension state (State 3), the stimulation remained the same as those in the swing flexion state for both legs except the right VI was activated at its saturation value; the stimulus pulse width values for the right SR, TF and TA were reduced to 0, while stimulus pulse width to the right IP was lowered to 75% of the saturation value. This change was initiated at the end of the swing flexion state and was active for 400 ms. After the step, the controller reentered clinical baseline standing mode (State 0) with the feet in tandem stance. The subject used his volitional UE effort with help from the experimenter to return the swing leg to the nominal position for comfortable erect bipedal standing in preparation for the next perturbation.

### Analysis

A total of 31 reactive steps were collected for analyis from two consecutive standing sessions. There was a practice period of 2 steps to begin the first session where very little ground clearance was achieved and there was no forward step progression. Stepping with the neuroprosthesis is an effort that requires coordination between the upper body and lower body stimulation. The subject required a few trials to get used to the experimental protocol and the lower body response to perform an effective step. These steps were not a part of subsequent analysis. After these initial practice steps, all perturbations resulted in reactive steps with forward foot progression. This resulted in 29 steps of data, 15 steps at 15% BW and 14 steps at 17.5% BW. Analysis of stepping results was performed using an unequal varience t-test to compare and contrast reaction timing and step length to different pull strengths. Table 2 describes each standing round, the session it was a part of, parameters of the experiment, and whether and how much data were analyzed.

**Table 2:** Overview of standing sessions, control types, and data collection

Standing Round	Session	Number of Steps	Perturbation	Control type	Purpose	Number trials analyzed
1–2	1		None	Feed Forward	Adjusting Activation Parameters	None
3	1		2–17.5% BW	Feed Forward	Observing Stepping Time	None
4	1		2–10% BW	Reactive	Adjusting Threshold Parameters	None
5	1		5–17.5% BW	Reactive	Adjust Threshold parameters	None
6	2	17	15% BW	Reactive	Experimental Procedure Practice and Data Collection	Last 15
7	2	14	17.5% BW	Reactive	Data Collection	All

## Results

The reactive stepping controller successfully detected body perturbations and appropriately modulated stimulation to lengthen the BOS so as to improve balance recovery. The controller detected perturbations above 10% BW every time and initiated a step.

**Fig. 6:**
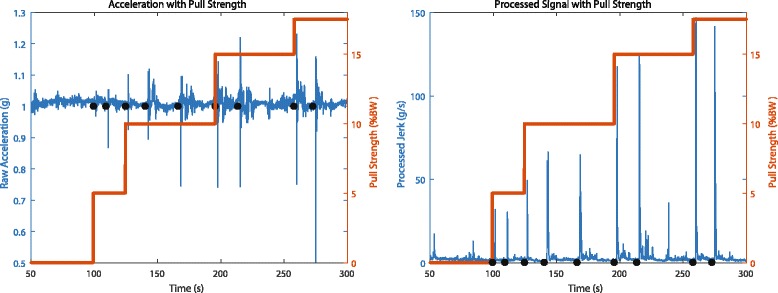
Raw accelerometer signal compared with the processed signal (both blue) for different pull magnitudes (orange). Black dots mark the location of actuator pulls

The combined jerk signal derived from the processed acceleration components enabled effective determination of pull magnitude of the peturbation applied to the whole body. Figure 6 shows a comparison between the raw accelerometer signal with the processed signal at different pull magnitudes. Note that while the raw accelerometer signal has maximum and minimum values that were not appreciably different between pull magnitudes of 10, 15, and 17.5% BW, the processed signal (jerk) defined by Eq. 1 clearly attained significantly different values for different pull magnitudes.

The dynamic responses by the subject with SCI have closely similar latencies when compared with those for able bodied subjects undergoing postural perturbations [16, 17] and are sumarized in Table 3. For all steps, the mean reaction time after perturbation onset was 137–280 ms for able-bodied individuals [17, 18] and 125 ms (S.D. 18) for the subject with SCI (measured as the crossing of T1 and the onset of stimulation). The average time between posture shifting with the ankles and hips and the beginning of leg unloading was 200 ms for able-bodied individuals [17] and 180 ms (S.D. 53) for the subject with SCI. The average time to unload the leg after perturbation onset was ranged from 473 to 610 ms (depending on age and experimental setup) [17, 18] for able-bodied individuals and 545 ms (S.D. 36) for the subject with SCI. The average time to complete the step with ground contact of the swing foot ranged from 614 to 780 ms (depending on age and experimental setup) for able-bodied individuals [16] and 750 ms (S.D. 69) for the subject with SCI. The mean step length was 163 mm (S.D. 59), around half the length of able-bodied adults (290–371 mm [18]).

**Table 3:** Comparison of reactive step timing components between the subject with SCI and able-bodied individuals

	Subject with SCI		Able-Bodied Individuals	
	Incr. Time (S.D.)	Total Time (S.D.)	Incremental Time	Total Time
First Reaction	125 (18)	125 (18)	137–280 [17, 18]	137–280 [17, 18]
Begin Unloading	180 (53)	305 (64)	200 [17]	480 [17]
Foot Off Ground	240 (64)	545 (36)	130 [17]	473–610 [17, 18]
Foot On Ground	205 (59)	750 (69)	140–170 [16, 18]	614–780 [16–18]

With the processed accelerometer feedback signal, the use of multiple thresholds enabled detection of larger perturbations more quickly than for smaller perturbations (*p* < .005). This difference in time was even more prominent for the second threshold crossing than the first. The average T1 crossing for perturbations of 15% BW was 133 ms (S.D. 67) and for 17.5% BW it was 114 ms (S.D. 58). For T2 crossing, the average time to threshold for 15% BW was 210 ms (S.D. 67), while the average for 17.5% BW was 163 ms (S.D. 58). This faster detection for larger perturbations produced a shorter prestep period, a faster leg lift, and faster touchdown, which are sumarized in Table 4. There was no significant diffence in step length between the two experimental conditions.

**Table 4:** Comparison of reactive step timing components between 15 and 17.5% BW pulls

	15% BW Pulls		17.5% BW Pulls	
	Incr. Time (S.D.)	Total Time (S.D.)	Incr. Time (S.D.)	Total Time (S.D.)
First Reaction	133 (18)^**^	133 (18)^**^	114 (13)^**^	114 (13)^**^
Begin Unloading	202 (68)	327 (79)	167 (30)	282 (31)
Foot Off Ground	236 (77)	563 (27)^**^	243 (49)	525 (34)^**^
Foot On Ground	220 (50)	783 (49)^*^	190 (65)	715 (71)^*^

^*^ *P* < 0.01; ^**^ *P* < 0.005

## Discussion

The control system described represents the first reactive controller applied to a neuroprosthesis with the goal of improving standing stability by altering the base of support. By utilizing body-mounted accelerometers on the subject’s waist and thigh, the perturbation magnitude was reliably detected. The two-phase reactive stepping controller was able to realize a complete protective step following perturbations in approximately the same amount of time as the intact central nervous systems (CNS).

The total time for completing the step after a perturbation with neural stimulation fell within the range of those reported for the intact CNS for cable-pull experiments (750 ms versus 614–780 ms [16–18]). Differences in step timing from reported values could be a compounded result of differences in perturbation waveform (weight drop, position controlled actuator, force controlled actuator), age, instructions given to the subjects, and the predictability of the perturbation. Despite these potential confounding factors, all the reactive steps are effective in stabilizing posture in response to external cable pulls. Though the total time to complete the step was similar between the able-bodied experiments and the FNS system, different components of the step had very different latencies. The reaction time and beginning of unloading the leg was faster with FNS while the time to actually pick up the leg and complete the swing phase was slower.

The ability of the FNS system to detect that a disturbance is occurring is similar to a healthy young adult and faster than older adults when comparing the first muscle response after the perturbation [18]. This reaction time translated into step preparation motion faster when compared to the reactive movements of an intact CNS [17]. This was due to the nature of the sensory systems used as feedback in the two cases. For the subject with SCI, control was based solely on signals obtained from accelerometers placed near the point of perturbation application. For an intact CNS, detection of destabilizing perturbations utilizes a complex combination of visual, vestibular, and proprioceptive influences distributed throughout the entire body. Though local feedback loops are able to provide some corrective strategies with very short latencies, to make a coordinated full body response, these different sensory systems must make individual estimates of body orientation and movement. Though this process takes more time than relying on a few single-purpose sensors, it allowed for greater flexibility in body positioning and orientation, voluntary movements before and during perturbations, and reliable detection of the perturbation direction. For the implanted standing neuroprosthesis, the choice of using the sum of the derivative of all of the accelerometer signals enabled quick and accurate detection of body perturbations, allowing for the triggering of a protective step faster than the latency inherent in the intact sensorimotor system.

Although it is theoretically possible to determine the exact magnitude and direction of the perturbation by measuring the accelerations of multiple body segments, this requires the use of more complex signal processing than the sampling rate of 40 Hz allows. The perturbation detection system described in this study employed a simplified jerk signal derived from a small set of accelerometers for determining the presence and magnitude of an applied perturbation. This method did not determine perturbation direction; however, the acceleration vectors (resultant magnitude and phase angles) calculated from each of the three signals may provide the ability to determine the direction of an applied disturbance. Future investigation will explore methods that will detect the direction of the perturbation in addition to the magnitude, enabling more complex control of step direction. We will also examine how a distributed sensor network can be used to detect the location of the perturbation, such as a support surface slip. These methods will look at ways to calibrate sensors after application, automatically determining the orientation of each sensor with respect to the body, and optimally processing the data from multiple non-ideal sensors with a Kalman filter or other data fusion techniques.

The goal of this work was to build a reactive stepping controller that could seamlessly integrate current and expected technologies for improved control of standing balance with an implanted neuroprosthesis. The use of three accelerometers and their locations in this study were based on our experience with similar sensors for determining the center of mass for bipedal standing balance [19, 20]. Other studies have shown effective detection of a fall with sensor placements on the lumbar and feet [21]. For this controller to be a feasible addition to current technologies and effective for use outside of the controlled laboratory environment, external sensors must be redesigned to be easily donned, doffed and recalibrated prior to each application, or implanted sensors need to be developed and deployed with the neural stimulation system. One new, scalable platform for implanted neuroprostheses consisting of stimulating and bipotential recording modules distributed where needed for a specific application throughout the body, known as the networked neuroprosthesis system (NNPS), is being developed by our group [22–25]. Each module in the NNPS contains a 3D accelerometer and gyroscope, thus fully integrating physical sensing from multiple locations into a fully implanted system. Such developments could obviate the necessity for external body mounted sensors and ultimately make out-of-the laboratory or at-home use of reactive stepping controllers possible in future clinical trials of standing neuroprostheses. Multiple, distributed implanted sensor sites could also enable development of advanced control systems other than reactive stepping, such as those that allow users to specify, assume and maintain non-erect or forward- and side-leaning postures for reaching tasks important for many activities of daily living while standing or seated in the wheelchair [26].

The time between initial reaction and beginning of unloading of the leg was approximately the same between the stimulated FNS system and the corresponding values observed in persons with an intact CNS [17]. The total time it took to unload the leg however was much slower with stimulation [16, 18]. This was likely due to excitation-contraction coupling delays and the latency inherent in recruiting muscle force with FNS. During clinical baseline standing in the subject with SCI who was recruited for the experiment described here, hip flexor muscles received no stimulation and the muscles were completely at rest. However, standing with an intact CNS has some low baseline activity for all muscles for facilitating finer control of balance [27]. Therefore, it may take less time for an intact CNS to generate a movement than it does for implant recipients with SCI, even when the commands were given at the same time. While the excitation-contraction coupling delays may be difficult to overcome, there are several techniques to increase the rate of force production, such as increased stimulus frequency or variable frequency pulse trains or initial doublet or triplet pulses that take advantage of the “catch property” of skeletal muscle and afford more rapid responses [28–30]. These strategies were not employed in this study, but should also be pursued in the future.

In addition to having a slower leg lift time, the time to complete a swing was slower with neural stimulation when compared to an intact CNS [16, 18]. One reason for this may be that no sensors were used to determine when the foot had achieved complete ground clearance. When a step was invoked after a perturbation, the amount of time it took to lift the foot varied with posture and the position of the center of mass. An intact CNS is capable of sensing when the foot has left the ground using proprioceptive information from the hip, knee and ankle joints in combination with loading information from the foot to expedite moving the swing leg forward and placing it down at a spot appropriate for the perturbation. However, with current neuroprostheses, the worst case scenario must be assumed, and therefore the maximum time to lift the foot must be used as the predetermined time for leg flexion in order to ensure proper clearance. This can contribute to a longer mean swing time since the leg may be in the air longer than absolutely necessary. Swing time with neural stimulation could be reduced by implementing ground contact and proximity sensors and adjusting the duration of stimulation to the hip flexors, as well as the terminal location of the foot depending on the nature of the disturbance. This would reduce the average swing time and improve stepping ability while still ensuring ground clearance. This aspect will be explored in future studies.

At 163 mm, the step length was about half the step length of able-bodied adults [18]. It is difficult to assess whether this smaller step would be insufficient for stabilization as it likely results from a combination of factors. Though the neuroprosthesis might not be providing enough stimulation to cause the foot to reach out far enough, the experimental setup and external support provide limits to the step excursion. With the ability of the subject to brace using the external support, the COM moves significantly less forward, resulting in reduced needed distance in the forward step. Even if the leg kinematics are the same, this will result in a smaller step. Future experiments will address this by looking more specifically at joint characteristics and adjust the stimulation setup to allow for larger translations in the COM.

Our investigation only considered stepping with the right leg, however if the current posture puts the majority of the person’s weight on their right leg, it would be quicker and perhaps more effective to initiate a compensatory step with the left leg instead. Similarly, choice of the stepping leg is highly likely to be dependent on the direction of the applied disturbance, in which case the system will need to further mimic the intact balance control system to decide on the appropriate strategy. Future investigations will develop and implement similar stepping for the left leg and be able to optimally select which leg will result in the fastest step and most effective reaction to a given perturbation, thereby further improving standing stability.

Although the controller reported here was effective at generating a reactive step in response to perturbations, the neuroprosthesis used to restore standing is still not self-stabilizing, requiring the users to lean on external devices with. The controller did not reduce the UE load or affect the comfort of the subject.. The subject must still exert substantial forces on the handles of the walker to maintain balance and ground clearance of the stepping foot, and the potential for falls still exists. No engineered control system will be able to prevent all falls in response to all external perturbations, just as the intact postural control system can fail when exposed to large, sudden perturbations resulting in falls even in able-bodied individuals. Nevertheless, the reactive stepping controller described here provides an important first step toward improving the safety of implanted neuroprostheses for use in standing after SCI.

## Conclusion

In this work, we developed, verified and characterized the performance of a reactive stepping controller that detected forward-directed body disturbances and initiated a compensatory step to stabilize standing in a recipient of an implanted standing neuroprosthesis with T4 motor complete paraplegia. A simplified feedback signal derived from three body-mounted accelerometers related to the jerk of the whole body COM successfully differentiated between applied force pulls of magnitudes ranging from 2 to 17.5% body weight, and provided robust and reliable inputs to a finite state machine that activated the appropriate postural responses and protective steps accordingly. The use of multiple thresholds applied to the combined jerk input signal successfully modulated state activation times between the anticipated postural adjustment and swing phases of the reactive stepping maneuver, resulting in faster compensatory steps for stronger perturbations than for weaker ones. The dynamic responses of the reactive stepping controller had very similar component time periods when compared with values for able-bodied subjects undergoing similar postural perturbations. This research marks progress towards developing and deploying control systems which can improve safety and independence for persons with spinal cord injuries by providing the ability to take protective steps before potentially dangerous destabilizing influences threaten their balance while standing with implanted neural stimulation.

## Appendix

**Table 5:** Standing stimulation pulse width and amplitude values

Muscle	Amplitude (mA)	Standing Pulse Width (μs)	Threshold Pulse Width (μs)	Saturation Pulse Width (μs)
Right vasti (R VS)	2.1	24	6	24
Left vasti (L VS)	2.1	100	32	100
Right semimembranosus/hamstrings (R HS)	20.0	250	7	250
Left semimembranosus/hamstrings (L HS1)	20.0	250	12	250
Left semimembranosus/hamstrings (L HS2)	20.0	70	1	70
Right gluteus maximus (R GM)	20.0	250	1	250
Left gluteus maximus (R GM1)	20.0	250	7	250
Left gluteus maximus (L GM2)	20.0	250	20	250
Right posterior adductor magnus (R PA)	20.0	0	6	250
Left posterior adductor magnus (L PA)	20.0	250	1	250
Right iliacus/psoas major (R IP)	8.0	0	15	20
Left iliacus/psoas major (L IP)	14.0	0	83	83
Right gastrocnemius/soleus (R GS)	1.4	65	37	65
Left gastrocnemius/soleus (L GS)	2.1	200	33	200
Right tibialis anterior (R TA)	20.0	0	7	26
Left tibialis anterior (L TA)	1.4	0	27	250
Right quadratus lumborum (R QL)	2.0	0	24	34
Left quadratus lumborum (L QL)	8.0	0	22	23
Right erector spinae (R ES)	2.0	0	25	75
Left erector spinae (L ES)	8.0	0	11	112
Right gluteus medius (R ME)	20.0	250	3	250
Right sartorius (R SR)	20.0	0	16	250
Right tensor fasciae latae (R TF)	20.0	0	1	25

## Abbreviations

APA: Anticipatory postural adjustment
BOS: Base of support
BW: Body weight
CNS: Central nervous system
COM: Center of mass
ES: Erector spinae
FNS: Functional neuromuscular stimulation
GM: Gluteus maximus
GS: Gastrocnemius/soleus
HS: Semimembranosus/hamstrings
IL: Iliacus/psoas major
ME: Gluteus medius
NNPS: Networked neuroprosthesis system
PA: Posterior portion of adductor magnus
QL: Quadratus lumborum
SCI: Spinal cord injury
ST: Sartorius
TA: Tibialis anterior
TF: Tensor fasciae latae
UE: Upper extremity
VS: Vasti
